# Medical interns’ training in family medicine at a district hospital and primary health care clinics in Middelburg, Mpumalanga

**DOI:** 10.4102/safp.v66i1.5844

**Published:** 2024-05-04

**Authors:** Lushiku Nkombua, Amir Rahimi

**Affiliations:** 1 Department of Family Medicine, Faculty of Health Sciences, University of Pretoria, Pretoria, South Africa; 2 Department of Health, Middelburg Hospital, Middelburg, South Africa

**Keywords:** medical interns, training, HPCSA, district hospital, family medicine, primary health care

## Abstract

Training of medical interns at the Middelburg district hospital has been introduced as part of the mandatory 6 months’ rotation in Family Medicine department since 2021. This report provides an overview of what has been attained in 2021 and 2022. It covers various aspects of the activities medical interns have been exposed to in the Middelburg hospital and the surrounding primary health care clinics.

**Contribution:**

Sharing experiences of family medicine training for medical interns in district hospitals is essential because the 6 months’ rotation is new for most family physician trainers, especially those in small hospitals and primary health care clinics. Taking into account the paucity of evidence on the topic, the report brings current information that supports that training medical interns in district hospitals and primary health care clinics prepares them to be comfortable and competent clinicians for the generalist work during the community service year ahead.

## Introduction

For many years, Middelburg District Hospital in Mpumalanga serves as the clinical learning centre for the training of the students of health professions under the aegis of the University of Pretoria, Family Medicine Department. Family medicine in Middelburg is the only specialist unit in the hospital and also is the main clinical unit for the Mpumalanga clinical training centres. The unit has been training Bachelor of Clinical and Medical Practice (BCMP) (Clinical associates) students since 2010, in house programme. The unit also accommodates elective students from most universities in South Africa and a few students from abroad.

There is a consistent regular elective from the physician’s assistants’ students from the University of Wisconsin in the United States (US). This has been ongoing since 2016 with the exception of 2020 and 2021 because of the coronavirus disease 2019 (COVID-19) restrictions. Middelburg clinical learning centre is also accredited for the in-house training of the registrars in family medicine. Five specialists have graduated from this site; they obtained their Master’s degrees in family medicine as well as the Fellowship from the College of Family Physicians (South Africa). Some of them are providing clinical leadership at the hospital and in the province of Mpumalanga. Since the implementation of the new medical interns training programme, Middelburg hospital and Witbank Hospital are considered as one training complex for family medicine in the Nkangala district of Mpumalanga. The interns spent 4 months in Witbank and 2 months in Middelburg. The latter allows for more exposure to the district health system through practice at the district hospital and the primary health care clinics. The latest assessment by the Health Professions Council of South Africa (HPCSA’s) interns training committee in August 2023, judged the family medicine training programme as excellent.

The clinical learning centre’s experience and involvement in training of other categories of health professionals have made a seamless integration of the medical interns in its activities. The family physicians and other clinicians at the hospital have long time ties with the local University of Mpumalanga^1,2^ in the prevention and management of non-communicable and communicable diseases.

## Contextualisation

The 2 months’ rotation at the district hospital is embedded in the 6 months of family medicine training where the majority (4 months) of the time is spent in the sister department at Witbank regional/tertiary hospital.^3^

Clear guidelines are laid down by the HPCSA for the family medicine training in general and the district rotation in particular.^3^ The domain of family medicine offers the interns the opportunity to manage a wide spectrum of conditions in undifferentiated patients. Among other conditions are communicable and non-communicable chronic diseases, palliative care, acute and non-acute conditions, preventive medicine, clinical forensic medicine, an so forth, the list is non-exhaustive. Also created are collaboration and interaction with other primary care health workers such as clinical associates, allied professionals, pharmacists and nurses.

Ultimately the medical intern is able to contribute to the management of patients who present at any primary care facility, for example primary health clinic, community health centre or the district hospital. In addition to assessing and managing patients at these levels, the interns are capacitated to identify patients whose conditions require a higher level of care and appropriately refer, in consultation with the supervisor. In case a patient is admitted to the district hospital for the treatment, the admitting intern follows up the patient’s progress until discharge, to ensure continuity of care. The same principle applies in the primary health care facilities where the medical interns see patients with acute or chronic conditions in a continuum of longitudinal care until the rotation ends. Local circumstances dictate the length of stay at the primary health care (PHC) clinic or the work in the district hospital.^4^

## Implementation

This report is based on the interaction the family physicians, the medical officers and the medical interns have had during the year 2021 and 2022 at Middelburg district hospital and the surrounding primary health care clinics.

The following are the activities undertaken with each group of medical interns.

### Orientation

On the first day, medical interns are orientated on the functioning and organisation of services at the district hospital and PHC clinics and their respective roles in the health system of South Africa. The interns are also introduced to the nursing units’ managers, the allied professionals, the pharmacists and other administration staff they may need help from during their rotation in the district hospital.

### Clinical exposure and learning

The district hospital is a composite structure where the medical intern is exposed to all types of patients in different clinical units: emergency room, operating theatre, maternity, child health and general outpatient. Outreach services to PHC clinics are organised on a weekly basis for each intern.^5^

After hours’ work is mainly delivered in the emergency room, also called casualty unit. From time to time, the intern may be called to operating theatres to be an assistant for emergency surgical procedures (mainly obstetrical).

### Supervision and collaboration

While the interns work under the supervision of senior medical officers and family physicians, they also work side by side with the clinical associates and the nursing personnel as well as the other elective students of healthcare sciences.^5^

To streamline the monitoring and evaluation of the work done by the medical interns during the 2 months, we have developed a few working tools using the 2020 guidelines set by the HPCSA regarding the domain of family medicine and primary care. The developed tools were made to be user-friendly; therefore, we framed them as checklists (copies available on request from the corresponding author).

The following checklists are introduced to the intern at the beginning of the rotation, and they are monitored and evaluated throughout the rotation and also at the completion of their time at the district hospital:

- Daily output to report on the clinical work and exposure to skills during working hours and after hours as applicable.
- Hand over and clinical discussion participation on a daily basis.
- Continuing professional development (CPD) presentations on identified topics as per the list from the HPCSA.
- Signature of attendance registers at each academic and clinical meeting.
- Overall global formative assessment by the supervisor(s) using the intern’s logbook.

Where appropriate, the data is also entered in the interns’ logbook provided by the HPCSA.^6^

## Discussion

During the 12 months of 2021, the cumulative total of 27 medical interns rotated through Middelburg hospital for a 2 months’ district hospital and primary healthcare training. Almost the same number in 2022 where 29 medical interns were received in Middelburg.

### Exposure and responsibilities of the medical interns in the district

Each intern had a logbook supplied by the HPCSA for the recording of the activities during this rotation. Additional to the logbook, the five checklists mentioned above were used to establish and evaluate work done by the interns under our supervision.

### Daily outputs

**FIGURE 1:**
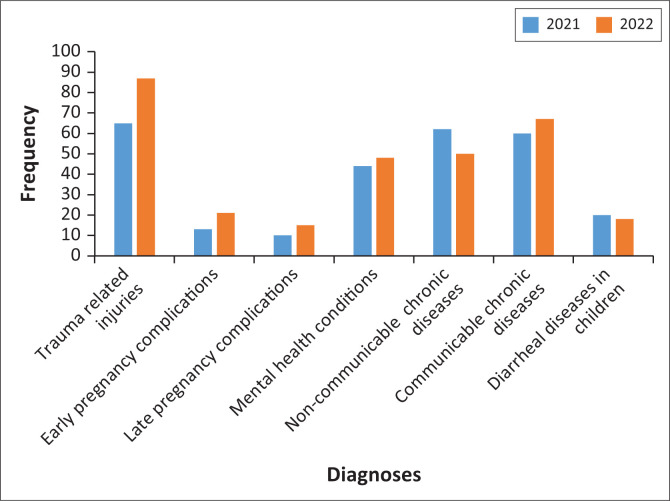
Morbidity reports.

The medical intern’s bulk of work in the district is mainly in the ambulatory care, primary health clinics, outpatients and the emergency room. The interns also have the opportunity to follow up patients they admitted to the wards for in-hospital treatment or for a short period of observation; this ensures the continuity of care. Admitted conditions vary from trauma-related injuries, interpersonal violence, complications of non-communicable diseases like hypertension, diabetes, asthma and epilepsy (see Figure 1). Human immunodeficiency virus (HIV)-related complications are also admitted. Sepsis, malnutrition and neonatal jaundice were found to be prominent in child health.^7^

The interns also documented high incidence of pregnancy-related complications especially bleeding in the first and the last trimesters. There are instances where interns took part in surgical operations as assistants to the surgeon (usually a senior medical officer or a family physician), mainly caesarean sections. In a few instances, the medial interns have assisted to the open reduction and internal fixation of fractures done by the visiting orthopaedic surgeons.

### Fast-forward to 2022

The year 2022 saw many of the health restrictions related to the COVID-19 pandemic being relaxed. The medical interns that started this year had more activities recorded than the previous ones, specifically in trauma-related care. It also included activities in line with community oriented primary care programme (COPC). The COPC activities were initiated by the family physicians with the support of the managers of the Hospital and PHC facilities. Similar initiatives have been reported by Singaram et al.^8^

**FIGURE 2:**
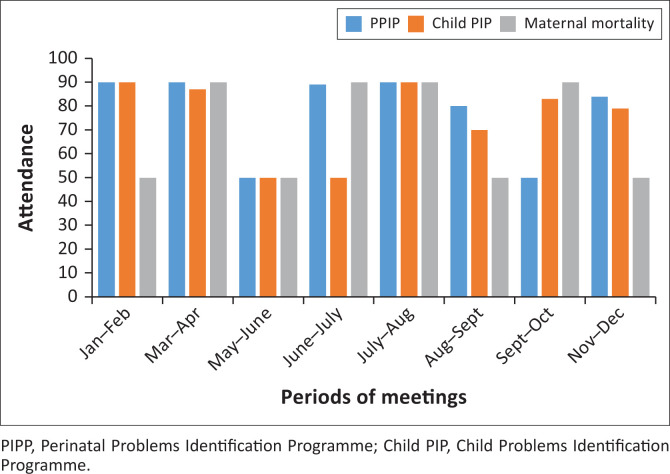
Attendance of interns to morbidity and mortality meetings.

A good balance is observed between the clinical exposure, learning and participation in the continuing professional development (see Figure 2). The latter includes monthly activities such as participation at the Perinatal Problems Identification Programme (PPIP), Child Problems Identification Programme (Child PIP) and other morbidity and mortality meetings. Interns are also encouraged to make presentations on topics of interest taking into account the diseases’ epidemiology of the area. A daily morning meeting takes place where interns and supervisors reflect on the previous day’s activities and also planning for the day ahead. A sizeable amount of learning takes place in these meetings.

Interns have been exposed to the broad spectrum of patients and conditions. Common conditions seen by interns reflected the burden of diseases in South Africa, mainly consisting of trauma-related injuries, adult communicable chronic illnesses, adult non-communicable chronic illnesses, maternal health issues including complications of pregnancy and childhood diarrhoeal diseases. Similar findings have been reported by Ross et al.^9^

Regarding theatre exposure, the medical interns did mostly attend to the gynaecological and obstetrical procedures; the most common operations were caesarean sections followed by laparotomies for ectopic pregnancy. Miller made similar observations elsewhere.^10^

## Recommendations

Medical interns’ training in the district hospital plays a pivotal role in that it provides an adequate platform for unselected patients and a variety of medical conditions. Both enhance learning and continuing professional development. The allocated period of 2 months for this rotation may not be enough to build confidence in many areas of the district health system. A recommendation to increase this period to 3 months would address this shortcoming.

## Limitations of this reflection

This report is a reflection from only one district hospital regarding a 2 months’ rotations in family medicine. It may not represent the situation in other hospitals in the province or in the country.

## Conclusion

Interns’ rotation in family medicine at the district hospital contributes to the strengthening of the district health systems especially supporting primary healthcare services in underserved areas. The rotation proved to develop competent clinicians who should contribute to the improvement of the population health and their wellbeing. Managers of health programmes and health facilities are called upon to support this rotation because it benefits the interns and the community at large.
